# Adult-Onset Still’s Disease: A Diagnostic Odyssey and Therapeutic Triumph

**DOI:** 10.7759/cureus.101399

**Published:** 2026-01-12

**Authors:** Niyas Khalid Ottu Para, Abdul Hakeem Muhammed, Seema Rab

**Affiliations:** 1 Internal Medicine, Burjeel Hospital, Abu Dhabi, ARE; 2 Internal Medicine, Burjeel Holdings, Abu Dhabi, ARE

**Keywords:** adult-onset still’s disease (aosd), hyperferritinemia, monocyclic still's disease, pyrexia of unknown origin (puo), yamaguchi syndrome

## Abstract

Adult-onset Still’s disease (AOSD) is a rare systemic autoinflammatory disorder defined by quotidian fevers, arthralgia, and marked hyperferritinemia, often mimicking infectious, autoimmune, or malignant conditions. We present a diagnostically challenging and clinically instructive case involving a 34-year-old man with persistent fevers, systemic inflammation, and hepatic dysfunction. Despite extensive investigation, infectious and autoimmune etiologies were excluded. The diagnostic breakthrough emerged upon identification of profound hyperferritinemia (6,900 ng/mL), prompting the diagnostic consideration of AOSD. Initiation of corticosteroid therapy led to rapid symptom resolution and biochemical normalization. This case underscores the vital role of pattern recognition, diagnostic exclusion, and timely immunosuppression in AOSD. We emphasize the importance of early ferritin assessment and highlight regional diagnostic biases, particularly among darker-skinned individuals, in whom rash may be absent or underrecognized.

## Introduction

Adult-onset Still’s disease (AOSD) is a rare, systemic autoinflammatory disorder characterized by quotidian high-spiking fevers, arthralgia or arthritis, hyperferritinemia, and multisystem involvement [1]. First described as the adult counterpart of systemic juvenile idiopathic arthritis, AOSD represents a distinct inflammatory entity with a highly variable clinical presentation [2]. The disease shows substantial overlap with infectious, malignant, and autoimmune conditions, which frequently complicates early recognition and leads to diagnostic delay [3].

The reported incidence ranges from 0.16 to 0.4 per 100,000 individuals, with prevalence estimates of 1-34 cases per million, underscoring its rarity and the limited clinical exposure most physicians have to this condition [4]. The absence of a disease-specific diagnostic test renders AOSD a diagnosis of exclusion [5]. Consequently, patients frequently undergo extensive and often prolonged investigations for fever of unknown origin (FUO), including broad infectious, oncologic, and rheumatologic evaluations, before a diagnosis is established [6]. This diagnostic delay may lead to unnecessary antimicrobial exposure, prolonged hospitalization, and delayed initiation of effective immunosuppressive therapy [7].

Classification systems such as the Yamaguchi criteria remain the most widely used framework for supporting the diagnosis once alternative etiologies have been rigorously excluded [8]. These criteria emphasize a constellation of clinical features and laboratory abnormalities rather than reliance on a single diagnostic marker. Alternative classification systems, including Fautrel criteria, incorporate hyperferritinemia and neutrophil predominance to improve diagnostic specificity [9].

Marked hyperferritinemia has emerged as a key biochemical hallmark of AOSD and serves as an important diagnostic clue in the appropriate clinical context [10]. Although elevated ferritin is nonspecific, extreme elevations, often exceeding 3,000-5,000 ng/mL, are highly suggestive of autoinflammatory syndromes, particularly AOSD, when infection and malignancy have been ruled out [11]. Hepatic involvement, manifested by transaminitis and cholestatic enzyme elevation, is also common and may further confound the diagnostic process by mimicking infectious or toxic hepatitis [12].

Recognition of AOSD may be especially challenging in regions where FUO is approached with a strong infectious bias, such as the Middle East, where zoonotic, viral, and parasitic infections are prevalent. Additionally, the characteristic evanescent salmon-colored rash, often considered a cardinal feature, may be subtle, transient, or difficult to appreciate in individuals with darker skin pigmentation, leading to underrecognition of the disease.

We present a diagnostically challenging case of AOSD with the classic triad of quotidian fever, arthralgia, and profound hyperferritinemia, accompanied by systemic inflammation and hepatic dysfunction in a 34-year-old young man from the United Arab Emirates. This case highlights the importance of systematic exclusion of mimicking conditions, the diagnostic value of ferritin trends, the limitations of relying on rash for diagnosis, and the dramatic therapeutic response to corticosteroids. Through this report, we aim to reinforce a structured approach to AOSD in patients with FUO and to emphasize the need for heightened clinical suspicion to enable timely diagnosis and treatment of this highly steroid-responsive yet frequently overlooked disease.

## Case presentation

**Figure 1:**
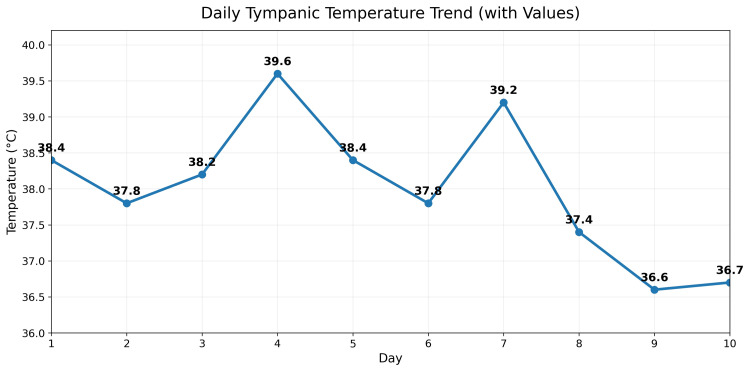
Spikes of fever

A 34-year-old previously healthy Asian man presented to the Emergency Department with a two-week history of persistent high-grade fever (Figure 1), diffuse arthralgia, and rapidly progressive fatigue. He reported that the fevers occurred with clockwork regularity, spiking to high-grade levels each evening, and were accompanied by aching pain affecting both large and small joints symmetrically, along with a sore throat. He denied weight loss, cough, gastrointestinal or urinary symptoms, night sweats, or contact with sick individuals. There was no recent travel, no ingestion of unpasteurized dairy, and no occupational or environmental exposure to livestock, untreated water, or zoonotic sources.

At presentation, he appeared visibly unwell, febrile, diaphoretic, and uncomfortable even at rest, with significant joint tenderness and restricted mobility due to pain. Despite the severity of systemic symptoms, he remained hemodynamically stable, alert, and oriented. Physical examination revealed no visible rash. It was explicitly noted that the patient's darker skin tone could render the classic evanescent erythema imperceptible, a recognized factor contributing to diagnostic delay in such patients. There was no peripheral lymphadenopathy or hepatosplenomegaly. Cardiopulmonary and abdominal examinations were unremarkable.

Initial laboratory evaluation, summarized in Table 1, revealed a marked systemic inflammatory response, with significantly elevated inflammatory markers. Liver biochemistry demonstrated a mixed hepatocellular and cholestatic pattern of injury, with elevated transaminases as well as raised cholestatic enzymes. Total and direct bilirubin levels were mildly elevated. Platelet counts were initially within the lower to normal range and subsequently increased during the hospital course. Renal function, lipid profile, and serum triglycerides were within normal limits.

**Table 1:** Summary of laboratory investigations during hospital course EBV: Epstein-Barr virus; CMV: cytomegalovirus; TB: tuberculosis; ANA: antinuclear antibody; dsDNA: double-stranded DNA; RF: rheumatoid factor; anti-CCP: cyclic citrullinated peptide; ENA: extractable nuclear antigen

Parameter	Result	Reference range
Inflammatory markers		
C-reactive protein	>240 mg/L	<5 mg/L
Erythrocyte sedimentation rate	24	<20 mm/hour
Ferritin	6,900 ng/mL	30-400 ng/mL
Liver biochemistry		
Alanine aminotransferase	126 U/L	<40 U/L
Aspartate aminotransferase	76 U/L	<40 U/L
Alkaline phosphatase	170 U/L	40-120 U/L
Gamma-glutamyl transferase	249 U/L	<60 U/L
Total bilirubin	22 µmol/L	5-21 µmol/L
Direct bilirubin	10.9 µmol/L	<5 µmol/L
Hematologic parameters		
Platelet count (initial)	160-170 × 10⁹/L	150-450 × 10⁹/L
Platelet count (subsequent)	>200 × 10⁹/L	150-450 × 10⁹/L
Peripheral blood smear	Normal	-
Metabolic and renal profile		
Serum triglycerides	1.4 mmol/L	<1.7 mmol/L
Serum cholesterol	3.82 mmol/L	<5.2 mmol/L
Renal function tests creatinine	78 µmol/L	64-104 µmol/L
Infectious workup		
Blood, urine, sputum cultures	Negative	-
Viral and zoonotic serologies		
Dengue	Negative	-
Chikungunya	Negative	-
Leptospirosis	Negative	-
Brucellosis	Negative	-
Typhoid	Negative	-
EBV	Negative	-
CMV	Negative	-
HIV	Negative	-
Hepatitis A/B/C	Negative	-
Malaria testing	Negative	-
Quantiferon-TB gold	Negative	-
Autoimmune panel		
ANA, dsDNA, RF, anti-CCP, and ENA	Negative	-
Complement levels	Normal	-

In keeping with a structured approach to FUO, a broad workup was initiated to systematically exclude infectious, malignant, and autoimmune mimics. Extensive cultures of blood, urine, and sputum remained sterile. Viral and zoonotic serologies, including dengue, chikungunya, leptospirosis, brucellosis, typhoid, Epstein-Barr virus, cytomegalovirus, human immunodeficiency virus, and hepatitis A, B, and C, were all negative. Malaria testing and tuberculosis screening using interferon-gamma release assay were nonreactive. High-resolution computed tomography of the chest, abdomen, and brain demonstrated no evidence of malignancy, granulomatous disease, or occult abscess formation.

An extensive autoimmune and rheumatologic evaluation was similarly unrevealing. Antinuclear antibody (ANA), anti-double-stranded DNA, rheumatoid factor (RF), anticyclic citrullinated peptide antibody, and extractable nuclear antigen panel were all negative. Complement levels were preserved. Hematologic evaluation showed no cytopenias, atypical lymphocytes, or blasts on peripheral smear. Serum ferritin, which had been pending during the early phase of evaluation, subsequently returned as markedly elevated.

Despite the initiation of broad-spectrum empiric antibiotic therapy with meropenem and linezolid, the patient continued to experience daily febrile spikes, and inflammatory markers remained persistently elevated. The presence of extreme hyperferritinemia in conjunction with systemic inflammation and hepatic dysfunction, in the absence of cytopenias or coagulopathy, raised a strong suspicion for an autoinflammatory syndrome, most notably AOSD.

**Figure 2:**
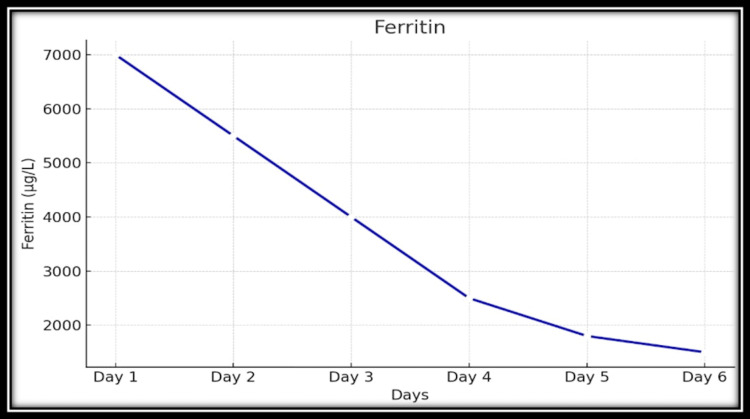
Trend showing a rapid decline in serum ferritin levels following initiation of corticosteroid therapy

High-dose systemic corticosteroid therapy was initiated empirically, guided by disease severity and hepatic involvement, with dexamethasone. The clinical response was rapid and striking. Within 24 hours, febrile episodes abated, and within 48 hours, the patient was afebrile, reporting substantial improvement in joint pain and fatigue. Inflammatory markers declined significantly; ferritin levels showed a marked downward trend; and liver enzyme abnormalities improved rapidly. Figure 2 illustrates the temporal relationship between corticosteroid initiation and ferritin decline in this case. The dramatic response to corticosteroid therapy served both therapeutic and diagnostic roles. The patient was subsequently transitioned to oral prednisolone with a structured taper. He remained in clinical remission at follow-up, with normalization of laboratory parameters and no recurrence of symptoms.

Investigations

A comprehensive and sequential investigative strategy was employed. Infectious disease evaluation included bacterial cultures from blood, urine, and sputum, all of which were sterile. Extensive serological testing excluded dengue, chikungunya, leptospirosis, brucellosis, typhoid, Epstein-Barr virus, cytomegalovirus, human immunodeficiency virus, and viral hepatitis. Malaria screening and tuberculosis testing using interferon-gamma release assay were negative. Cross-sectional imaging of the chest, abdomen, and brain excluded malignancy, abscess formation, and granulomatous disease.

Autoimmune investigations demonstrated no evidence of systemic connective tissue disease. ANA, anti-double-stranded DNA, RF, anticyclic citrullinated peptide antibody, and extractable nuclear antigen panels were all negative. Complement levels were preserved. Inflammatory markers were markedly elevated. Hematologic assessment revealed a normal complete blood count, with no cytopenias, blasts, or evidence of hemolysis. Lipid profile and coagulation parameters were within normal limits. The pivotal diagnostic clue was the identification of extreme hyperferritinemia, which, in the appropriate clinical context, strongly supported a diagnosis of AOSD.

Differential diagnosis

The diagnostic journey in this case was marked by the systematic exclusion of several high-priority differential diagnoses, each considered and ruled out on the basis of specific findings, as shown in Table 2. Ultimately, the differential narrowed to AOSD, supported by the triad of quotidian fevers, arthralgia, and hyperferritinemia, within the context of an otherwise negative diagnostic workup.

**Table 2:** Differential diagnoses considered and rationale for exclusion Alternative diagnoses considered during the diagnostic workup have been summarized, and the clinical, laboratory, and imaging findings that supported or argued against each condition have been outlined EBV: Epstein-Barr virus; CMV: cytomegalovirus; TB: tuberculosis; ANA: antinuclear antibody; dsDNA: double-stranded DNA; RF: rheumatoid factor; anti-CCP: cyclic citrullinated peptide; ENA: extractable nuclear antigen

Differential diagnosis	Key supporting features considered	Findings against diagnosis
Sepsis and occult bacterial infections	Persistent fever and systemic inflammation	Lack of clinical or biochemical response to empiric broad-spectrum antibiotics, with negative cultures and imaging
Viral illnesses	Fever with hepatic involvement	Negative viral serologies for EBV, CMV, HIV, and hepatitis A-C
Zoonotic and regional infections	Geographic exposure risk and prolonged febrile illness	Targeted testing for brucellosis, leptospirosis, typhoid, and tuberculosis was negative
Autoimmune rheumatic disease	Systemic inflammation with hepatic involvement	Negative ANA, RF, anti-dsDNA, anti-CCP, and ENA, with absence of suggestive clinical features (e.g., rash, oral ulcers, and serositis)
Malignancy	Fever of unknown origin with inflammatory markers	No lymphadenopathy, cytopenias, or abnormalities on cross-sectional imaging
Macrophage activation syndrome/hemophagocytic lymphohistiocytosis	Marked hyperferritinemia and systemic inflammation	Absence of cytopenias or hypertriglyceridemia and rapid clinical response to corticosteroids

Diagnosis

The diagnosis of AOSD was established based on the internationally accepted Yamaguchi criteria, in combination with a structured process of exclusion and a hallmark therapeutic response. The patient fulfilled five criteria, meeting the required threshold: two major (fever >39°C for greater than one week; arthralgia greater than two weeks) and three minor (abnormal liver function tests, negative ANA/RF, and leukocytosis >10,000/μL, which is present in an inflammatory context presented in Table 1). The classic rash was not observed, likely due to skin pigmentation; however, its absence did not preclude diagnosis, particularly given the growing literature supporting a diagnosis even when the rash is subtle or unapparent in darker-skinned individuals. The hyperferritinemia (>6,900 ng/mL) provided additional support for the diagnosis, although not included in the original criteria. The rapid and reproducible response to corticosteroids further served as a strong diagnostic confirmation. Although glycosylated ferritin was unavailable for testing, the case still aligned with several features of the Fautrel criteria. Exclusion of all mimicking conditions, fulfillment of classification criteria, and therapeutic validation via steroid response provided a robust foundation for diagnosis.

Treatment and outcome

Empirical high-dose corticosteroids were initiated based on strong clinical suspicion of AOSD following exclusion of other causes. The patient received intravenous dexamethasone 6 mg twice daily, approximately equivalent to 75 mg of oral prednisolone. Within 24 hours of initiation, the fever curve flattened, and by 48 hours, the patient was afebrile and reported marked improvement in systemic symptoms and joint pain. Inflammatory markers declined rapidly: C-reactive protein halved, ferritin dropped to 3,700 ng/mL, and liver enzymes normalized. The patient was transitioned to oral prednisolone 60 mg daily, with a six-week taper (60→50→40→30→20→10→5 mg). He remained afebrile and symptom-free throughout tapering. Outpatient follow-up showed continued clinical and biochemical remission. Given his response and complete recovery, the patient was classified as having a monocyclic pattern of AOSD. He was educated about relapse signs and referred to rheumatology for surveillance. No steroid-sparing agents were required at the time of writing.

## Discussion

AOSD is an uncommon systemic autoinflammatory disorder that continues to challenge clinicians because of its heterogeneous presentation, lack of a definitive diagnostic test, and substantial overlap with infectious, malignant, and autoimmune conditions [1]. As emphasized in the Introduction section, AOSD remains a diagnosis of exclusion, and delays in recognition are common, particularly in patients presenting with prolonged fever and markedly elevated inflammatory markers. The present case illustrates a classic yet diagnostically complex presentation of AOSD and underscores the importance of a structured, criteria-based approach supported by longitudinal laboratory trends and therapeutic response.

The patient presented with prolonged daily fever, diffuse arthralgia, striking hyperferritinemia, and hepatic dysfunction, features that immediately raised concern for severe infection, inflammatory liver disease, hematologic malignancy, or a hyperinflammatory syndrome such as macrophage activation syndrome (MAS). Such diagnostic dilemmas, in which rare conditions closely mimic more common pathologies, are well recognized in clinical practice and have been similarly documented in uncommon central nervous system tumors, such as hemangioblastoma, that can masquerade as infectious or metastatic disease [6]. Consistent with the diagnostic framework outlined in the Abstract and Investigation sections, an extensive and systematic evaluation was undertaken. Broad microbiological testing encompassing bacterial cultures, viral serologies, zoonotic infections, mycobacterial disease, and regionally relevant pathogens was uniformly negative. Similarly, comprehensive autoimmune and rheumatologic testing, including ANA, RF, anti-cyclic citrullinated peptide antibody, anti-double-stranded DNA, and extractable nuclear antigen panel, failed to identify an alternative connective tissue disease. Cross-sectional imaging of the chest, abdomen, and brain excluded occult malignancy, abscess formation, granulomatous disease, and lymphoproliferative disorders. The absence of sustained response to broad-spectrum antibiotics further supported a noninfectious inflammatory etiology.

Within this context, the application of validated classification criteria was central to establishing the diagnosis. The Yamaguchi criteria, which remain the most widely used classification system for AOSD, are central to diagnosis, which requires the presence of at least five criteria, including at least two major criteria, after exclusion of infections, malignancy, and other rheumatic diseases [8]. This patient met two major criteria (prolonged high fever, arthralgia) and three minor criteria (hepatic dysfunction, negative RF/ANA, leukocytosis), thereby meeting the required threshold of five or more criteria after rigorous exclusion of alternative diagnoses. Although the characteristic evanescent salmon-pink rash was not clinically appreciated, its absence does not preclude the diagnosis. Published series indicate that a significant minority of patients do not exhibit a clearly documented rash, and this limitation may be amplified in individuals with darker skin pigmentation, in whom transient erythema can be difficult to recognize [12]. Taken together, the patient met the threshold for classification as AOSD according to Yamaguchi criteria once mimicking conditions had been rigorously excluded.

Alternative classification systems further support the diagnosis. The Fautrel criteria incorporate hyperferritinemia and low glycosylated ferritin as diagnostic elements and do not require exclusion of other diseases [9]. Although glycosylated ferritin testing was not available in this case, the presence of markedly elevated total ferritin, quotidian fever, arthralgia, neutrophil predominance, and negative autoimmune serology aligns closely with the Fautrel framework. Importantly, the lack of access to specialized biomarkers such as glycosylated ferritin or interleukin-18 reflects real-world limitations faced by many centers and reinforces the continued relevance of clinical criteria and response to therapy in diagnosing AOSD [9].

Hyperferritinemia (6,900 ng/mL) was the pivotal biomarker that redirected the diagnostic framework. In the context of a negative infectious workup, such an extreme elevation is a powerful signal for MAS, principally AOSD in this setting, and justified the empirical trial of immunosuppression [10,11]. While elevated ferritin is nonspecific, levels exceeding 3,000-5,000 ng/mL are highly suggestive of AOSD when infectious and malignant causes have been excluded [11]. The patient’s ferritin peaked at 6,900 ng/mL and subsequently declined in parallel with clinical improvement and corticosteroid therapy, providing strong supportive evidence for the diagnosis. Serial ferritin measurements proved more informative than isolated values, allowing dynamic assessment of disease activity and therapeutic response [11].

Hepatic involvement was a prominent feature and significantly contributed to diagnostic complexity. Liver enzyme abnormalities are reported in a majority of patients with AOSD and may range from mild transaminitis to severe hepatitis [12]. In this patient, the degree of aminotransferase elevation initially raised concern for infectious or toxic hepatitis. However, the rapid improvement in liver enzymes following corticosteroid escalation, along with parallel improvements in ferritin and systemic inflammation, strongly suggests immune-mediated hepatic involvement as part of the AOSD disease spectrum [12]. The transient rebound in transaminases observed during early steroid taper further highlights the inflammatory nature of the hepatic dysfunction and the importance of adequate induction dosing.

MAS was carefully considered, given the extreme hyperferritinemia. MAS is a well-recognized and potentially fatal complication of AOSD and requires early identification [7]. However, several features argued strongly against MAS in this case, including preserved platelet counts, absence of cytopenias, normal triglyceride levels, normal to elevated fibrinogen, normalization of lactate dehydrogenase, and a sustained downward trend in ferritin. Moreover, the patient’s clinical stability and rapid improvement with corticosteroids were inconsistent with evolving MAS, which typically demonstrates progressive clinical deterioration despite standard therapy [7].

The therapeutic course further reinforces the diagnosis. Systemic corticosteroids remain first-line therapy for AOSD and induce remission in a substantial proportion of patients [5]. The dramatic resolution of fever and arthralgia, coupled with rapid biochemical improvement following high-dose corticosteroid therapy, represents a classic ex juvantibus confirmation of AOSD. This case also illustrates a key management principle: premature or excessive tapering of corticosteroids can lead to biochemical relapse, particularly in patients with hepatic involvement and high inflammatory burden. Subsequent stabilization with escalation to an appropriate prednisolone dose and gradual taper underscores the need for individualized treatment strategies guided by both clinical and laboratory parameters [5].

In the broader context of the literature, this case is consistent with published series describing AOSD as a major cause of FUO accompanied by hyperferritinemia and liver enzyme abnormalities [2]. Diagnostic delay is frequently reported, often due to initial attribution to infection or malignancy and overreliance on the presence of rash [12]. This case adds to the growing body of evidence that AOSD may present without overt cutaneous manifestations and that clinicians should maintain a high index of suspicion when confronted with compatible systemic features and laboratory abnormalities.

In summary, this case highlights the diagnostic value of a structured, criteria-based approach to AOSD, integrating systematic exclusion of mimicking conditions, application of established classification criteria, careful interpretation of ferritin and liver enzyme trends, and assessment of therapeutic response. Early recognition and appropriate immunosuppressive therapy resulted in rapid clinical improvement and a favorable short-term outcome. Increased awareness of AOSD and its variable presentation is essential to avoid diagnostic delay and to optimize outcomes in patients presenting with prolonged fever and systemic inflammation.

Clinical subtype and prognosis

AOSD manifests in three clinical patterns as suggested in Table 3. This patient’s full recovery, biochemical normalization, and steroid taper success indicate a monocyclic systemic pattern associated with the most favorable prognosis. Nonetheless, long-term surveillance is essential due to relapse potential. The patient will be followed in the rheumatology clinic periodically to monitor for relapse or evolution to a polycyclic or chronic articular pattern.

**Table 3:** Clinical subtypes of adult-onset Still's disease The recognized clinical course patterns of adult-onset Still’s disease and their characteristic features have been summarized Source: Adapted from previously published descriptions of disease course classification from [13]

Disease course pattern	Description
Monocyclic	A single systemic inflammatory episode followed by complete and sustained remission
Polycyclic	Recurrent inflammatory flares interspersed with periods of remission
Chronic articular	Persistent, progressive inflammatory arthritis resembling rheumatoid arthritis

Regional implications

In Middle Eastern clinical settings, the diagnostic approach to FUO often prioritizes infectious etiologies such as tuberculosis, brucellosis, and other endemic or zoonotic diseases. While this strategy is appropriate given regional epidemiology, it may inadvertently delay consideration of autoinflammatory disorders such as AOSD. This case highlights the importance of incorporating serum ferritin early in the evaluation of prolonged fever, particularly when initial infectious and autoimmune investigations are unrevealing. It also underscores that the absence of a characteristic rash should not exclude AOSD from consideration, especially in individuals with darker skin pigmentation, where subtle erythema may be difficult to appreciate. Finally, the dramatic and sustained response to corticosteroid therapy in this patient reinforces the diagnostic value of steroid responsiveness in appropriately selected cases, emphasizing the need for heightened clinical awareness of AOSD as a treatable cause of prolonged systemic inflammation in the region.

Final insight

This case not only fits established diagnostic frameworks but also highlights key real-world diagnostic challenges: racial dermatologic bias, infectious disease over-prioritization, and underuse of ferritin. Its instructive value lies in showcasing how methodical exclusion and biomarker integration can lead to timely, life-changing intervention.

## Conclusions

This diagnostic odyssey underscores that in a patient with quotidian fever and systemic inflammation, a persistently negative workup should prompt immediate ferritin assessment. Profound hyperferritinemia is a critical clue to AOSD, even in the absence of classic rash, and warrants a timely diagnostic-therapeutic trial of corticosteroids. AOSD should be considered when extensive infectious, autoimmune, and malignant evaluations are unrevealing, even in the absence of characteristic cutaneous findings. Recognition of hyperferritinemia as a key diagnostic clue, combined with careful exclusion of mimicking conditions, enabled timely diagnosis and treatment in this patient. The rapid and sustained response to corticosteroid therapy underscores both the diagnostic and therapeutic value of early intervention. Increased clinical awareness of atypical presentations of AOSD is essential to avoid diagnostic delay and to improve patient outcomes.
